# Whitefly predation and extensive mesonotum color polymorphism in an *Acletoxenus* population from Singapore (Diptera, Drosophilidae)

**DOI:** 10.3897/zookeys.725.13675

**Published:** 2017-12-29

**Authors:** Wong Jinfa, Foo Maosheng, Hugh T. W. Tan, Rudolf Meier

**Affiliations:** 1 Department of Biological Sciences, National University of Singapore, Singapore 117543; 2 Lee Kong Chian Natural History Museum, National University of Singapore, Singapore 117377

**Keywords:** *
Acletoxenus
*, Diptera, Drosophilidae, predatory maggot, Singapore, whitefly

## Abstract

*Acletoxenus* is a small genus of Drosophilidae with only four described species that are closely associated with whiteflies (adults and larvae). Here, the first video recordings of larvae feeding on whiteflies (*Aleurotrachelus trachoides*) are presented. Typical morphological adaptations for predation by schizophoran larvae are also described: the larval pseudocephalon lacks a facial mask and the cephaloskeleton is devoid of cibarial ridges that could be used for saprophagy via filtration. Despite being a predator, *Acletoxenus* is unlikely to be a good candidate for biological control of whiteflies because the life cycle is fairly long (24 days), lab cultures could not be established, and the puparia have high parasitization rates by a pteromalid wasp (*Pachyneuron leucopiscida*). Unfortunately, a confident identification of the Singapore *Acletoxenus* population to species was not possible because species identification and description in the genus overemphasize coloration characters of the mesonotum which are shown to be unsuitable because the Singapore population has flies with coloration patterns matching three of the four described species. Based on morphology and DNA sequences, the population from Singapore is tentatively assigned to *Acletoxenus indicus* or a closely related species.

## Introduction

Drosophilidae contains >3950 described species in 77 genera and two subfamilies (Bächli 2015). The best-known species is *Drosophila melanogaster* which is typical for most in the family in that it has saprophagous larvae. However, the larvae of many other drosophilid species utilize a wide variety of substrates and the natural history of the family is full of surprising convergence. For example, associations between drosophilid larvae and spittlebugs have evolved at least three times (Thompson and Mohd-Saleh 1995) and gave rise to a species-rich clade with more than 100 species (*Cladochaeta*: (Wheeler and Patterson 1952, Grimaldi and Nguyen 1999). Many other drosophilid species have larvae that prey on eggs, including the species in the *Drosophila simulivora* species group whose aquatic larvae feed on the eggs and larvae of Simuliidae, Chironomidae, and Odonata (Aubertin 1937, Tsacas and Disney 1974). Another case of surprising convergence is found in Steganinae. *Rhinoleucophenga* (Steganinae) and *Acletoxenus* have larvae that are predators of Sternorrhyncha (Malloch 1929, Clausen and Berry 1932, Ashburner 1981, Parchami-Araghi and Farrokhi 1995, Culik and Ventura 2009, Lambkin and Zalucki 2010, Yu et al. 2012). Yet, *Rhinoleucophenga* and *Acletoxenus* are distantly related; i.e., larval predation of Sternorrhyncha by steganine larvae likely evolved twice.

**Figure 1.**
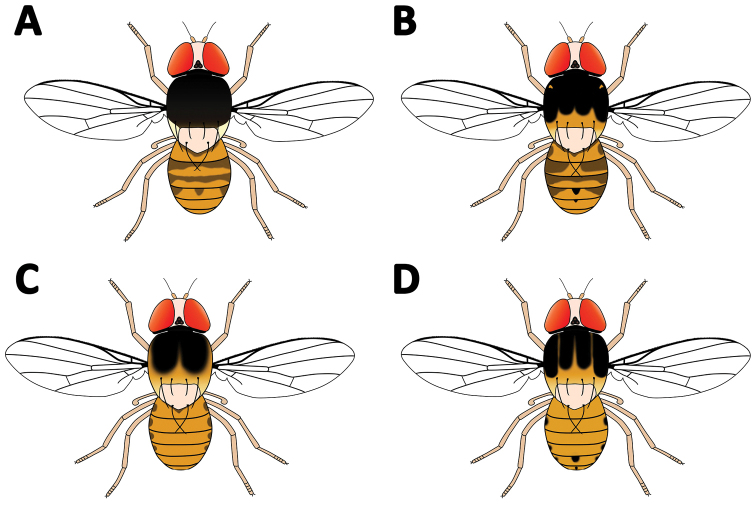
Morphology of **A** *Acletoxenus formosus* **B** *A. indicus* **C** *A. meijerei*, and **D** *A. quadristriatus*.

Recently, an *Acletoxenus* population was discovered in Singapore that was associated with whiteflies feeding on chilli plants, *Capsicum annuum* L. (Solanaceae). The population was studied in greater detail and we present the first video recordings documenting larval predation , provide a larval description, and determine the length of the life cycle. Lastly, we comment on the inappropriateness of using mesonotum coloration for species identification and description in *Acletoxenus*. The color patterns of the mesonotum are shown to be very variable within a single population. Yet, the description and identification of the four currently accepted species rely quite heavily on color pattern and chaetotaxy characters (Table 1, Fig. 1). This is partially due to the fact that the type of one of the species is female (*Acletoxenus indicus* Malloch, 1929) so that a comparison of male genitalia with the remaining species cannot be carried out. Fortunately, male type material is available for *Acletoxenus formosus* (Leow, 1864) (see Bächli 1984), *Acletoxenus quadristriatus* Duda, 1936, and *Acletoxenus meijerei*. The latter has syntypes in Berlin (Bächli 1984: sex not specified) and a male syntype in Amsterdam (Bächli 1987: now Leiden), but the location of the latter is currently unknown (Pasquale Cliliberti, pers. comm.).

**Table 1.** Morphological differences between the described species of *Acletoxenus*.

*Acletoxenus formosus* (Leow, 1864)	Proclinate orbital bristles not noticeably shorter than the anterior reclinate bristles (Malloch 1929)	Mesonotum almost entirely black with yellowish tan lateral margins (Malloch 1929, Bock 1982)
	Proclinate orbital bristles noticeably shorter than anterior reclinate bristles (Bächli et al. 2004)	
*Acletoxenus indicus* Malloch, 1929	Proclinate orbital bristles noticeably shorter than anterior reclinate bristles (Malloch 1929)	Mesonotum with central black vitta and two vittas on each side that are interrupted at suture and extend sublaterally (Malloch 1929)
*Acletoxenus meijerei* Duda, 1924	Proclinate orbital bristles not noticeably shorter than anterior reclinate bristles (Duda 1924, Malloch 1929)	Mesonotum with two broad dark vittas which are more or less confluent behind the suture and do not extend to the hind margin margin (Duda 1924, Malloch 1929, Bock 1982)
*Acletoxenus quadristriatus* Duda, 1936	Proclinate orbital bristles noticeably shorter than the anterior reclinate bristles (Malloch 1929)	Mesonotum with four broad dark longitudinal vittas coalescing or slightly separated, with the medial vittas reaching to rear third while the lateral ones almost to the posterior dorsocentral (Malloch 1929, McEvey 2016)

## Materials and methods

### *Acletoxenus* recruitment, collection, and identification

Chili (*Capsicum annuum* ‘Yang Jiao’) were grown along a building corridor of Block S2 of the Kent Ridge campus of the National University of Singapore (1°17'45.01"N, 103°46'41.08"E). Whiteflies naturally appeared on the chilli plants which in turn attracted *Acletoxenus*. Adult flies were captured and either stored in 100% ethanol or flash frozen with liquid nitrogen before being stored in a freezer at −80 °C. Three morphotypes were identified based on the pigmentation pattern of the mesonotum. These morphotypes corresponded to the descriptions and figures (see McEvey 2016) of *Acletoxenus formosus, Acletoxenus indicus*, and *Acletoxenus quadristriatus* (Malloch 1929, Duda 1936, Bock 1982). The relative abundance of the three morphotypes was determined, and Fisher’s exact probability 2 × 3 test was used to test whether the differences were significant. Samples were also sent to Dr. Gerhard Bächli from the Zoological Museum of the University of Zurich and Dr. Shane McEvey from the Australian Museum for identification. Samples of the whiteflies' fourth instars were sent to Dr. Paul De Barro (CSIRO).

### DNA barcoding

Genomic DNA was extracted from whole specimens of using QIAGEN DNeasy Blood & Tissue Kits. Polymerase chain reaction (PCR) was used to amplify the target cytochrome c oxidase, subunit I (COI) gene using primer pairs (Table 2). The PCR mixture (20 μL) contained 2.5 μL of buffer, 2 μL of dNTP, 1 μL of each primer of a primer pair, 0.15 μL of Ex Taq and 5 μL of template DNA. The program consisted of 40 cycles of amplification (30 sec of denaturation at 94 °C, 30 sec of annealing at 52 °C and 1 min of extension at 72 °C). The PCR products were then purified using BIOLINE SureClean according to the manufacturer’s protocol before cycle sequenced using BigDye Terminator ver. 3.1 Cycle Sequencing Kit. The cycle sequencing mixture (10 μL) contained 2 μL of buffer, 0.5 μL of BigDye, 1.75 μL of each primer and 2 μL of template DNA. The program consisted of 1 min of initial denaturation at 95 °C, followed by 25 cycles of amplification (30 sec of denaturation at 94 °C, 30 sec of annealing at 52 °C and 4 min of extension at 60 °C). An ABI 3730xl sequencer was used for sequencing. Reference COI sequences for *Acletoxenus formosus* (700 base pairs) and *Acletoxenus indicus* (1536 base pairs) were downloaded from GenBank (accession numbers EF576933, HQ701131). The sequences for the different *Acletoxenus* morphotypes from Singapore were then aligned against the reference sequences from GenBank using MAFFT ver. 7 using the default settings (Katoh and Standley 2013). Afterwards, MEGA6 was used to add the new sequences for *Acletoxenus* in order to determine pairwise distances (Tamura et al. 2013)

**Table 2.** Primer pairs used in PCR reaction.

Species	Primer name	Primer sequence
*Acletoxenus* (2 individuals from each sex and morphotype)	LCO1490	5’-GTCAACAAATCATAAAGATAT TGG-3’
	HCO2198	5’-TAAACTTCAGGGTGACCAAAAAATCA-3’
	s2183	5’-CAACATTTATTTTGATTTTTTGG-3’
	a3014	5’-TCCAAT GCACTAATCTGCCATATTA-3’
Whitefly prey	mlCOIintF	5’-GGWACWGGWTGAACWGTWTAYCCYCC-3’
	jgHCO2198	5’-TAIA CYTCIGGRTGICCRAARAAYCA-3’
Parasitoid wasp	LepF	5’-ATTCAACCAATCATAAAGATATTGG-3’
	LepR	5’-TAAACTTCTGGATGTCCAAAAAATCA-3’

### Are *Acletoxenus* predators?

Behavioral observations of *Acletoxenus* larvae and adults were made in the field and ex-situ. The ex-situ observations were based on individuals that were placed on whitefly infested leaves under a dissection microscope. Behavior was video-taped using a Canon LEGRIA HF S30 video camera. In addition, the morphology of the larvae and adults was studied in order to determine whether the species has features that are known to be typical of predatory larvae. For comparative purposes, the larvae of a known saprophage, *Drosophila melanogaster*, were also studied. All larvae were killed in hot soapy water before dehydration via a graded ethanol series (see Meier 1995, 1996). In order to study the cephaloskeleton, the larvae were cut at the mid-section and soaked in potassium hydroxide for 15 minutes (light microscopy with Olympus BX51) or three days (confocal microscopy: mounted on glass slide with Euparal; imaging with a Zeiss LSM 510 META at 20× using 488 nm wavelength with LP505 filter). The confocal images were rendered into a three-dimensional model with Amira 5.3.3.

### Life cycle of *Acletoxenus*

Field observations were used for determining the length of the life cycle of *Acletoxenus* because attempts to rear the species under laboratory conditions failed. Individual larvae on chili plant leaves infected with whiteflies were regularly tracked. Upon discovery of an *Acletoxenus* egg, larva, or puparium, its length was measured with Vernier calipers and the leaf was labelled. On the following day, all labelled leaves were checked for the presence of the same individual as determined by stage and size. If a larva was no longer present, the leaves in closest proximity were checked until a larva was located. The larva was deemed to be the same individual if its length was the same or slightly longer. All larvae that could no longer be located were excluded from determining the duration of the larval stage. If there were multiple larvae on a leaf, data were only collected if the lengths of the larvae were sufficiently different to distinguish individuals.

In order to determine adult longevity, adult emergence was documented by collecting puparia (n= 34) and placing them on moist tissue paper in an enclosed plastic container. Emergence was recorded with a Canon LEGRIA HF S30 video camera (see above). Newly emerged *Acletoxenus* were then used to determine the life span of adults by maintaining them in Petri dishes in an air-conditioned laboratory at 25 °C. The Petri dishes contained a piece of whitefly-infested leaf placed on a moist piece of tissue paper and a cotton ball soaked in honey. The leaves were changed every other day and the cotton ball weekly to ensure an adequate supply of food. The lifespan of each adult was calculated by counting the number of days from emergence to death.

### Parasitism

In the last four months of the experiment, the population of *Acletoxenus* declined and many *Acletoxenus* puparia were black instead of green. Parasitization was suspected and a few dark puparia were subsequently placed on wet tissue paper in a plastic container. Parasitoids emerged and were killed in 100% ethanol before identifying them using taxonomic keys (Mani 1939, Gupta and Poorani 2009). Photographs of the parasitoid wasp were also taken with a Nikon EOS-1 camera (Visionary Digital). Only empty parasitized puparial cases retained some dark brown pigments, which allowed for determining of the monthly rate of parasitism based on empty puparia (May–July 2014).

## Results and discussion

### No confident species identification despite a wealth of knowledge

**Figure 2.**
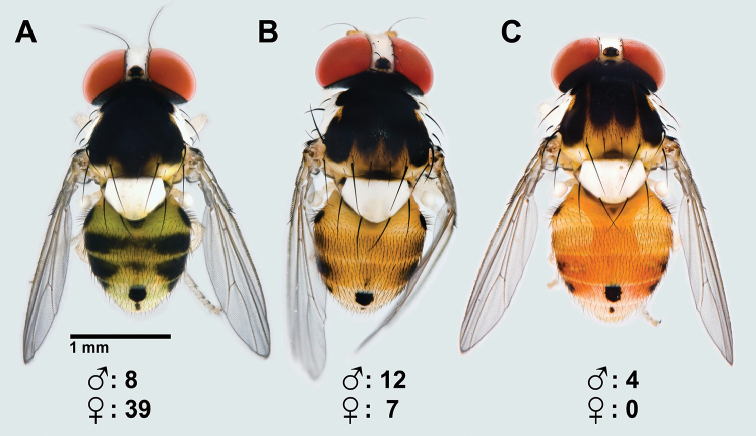
Mesonotum color patterns **A** entirely black **B** with central black vitta that is split and connected to two other vittas on each side, and **C** four dark longitudinal stripes; all three morphotypes were bred from larvae collected together on the same host plant in Singapore.

**Figure 3.**
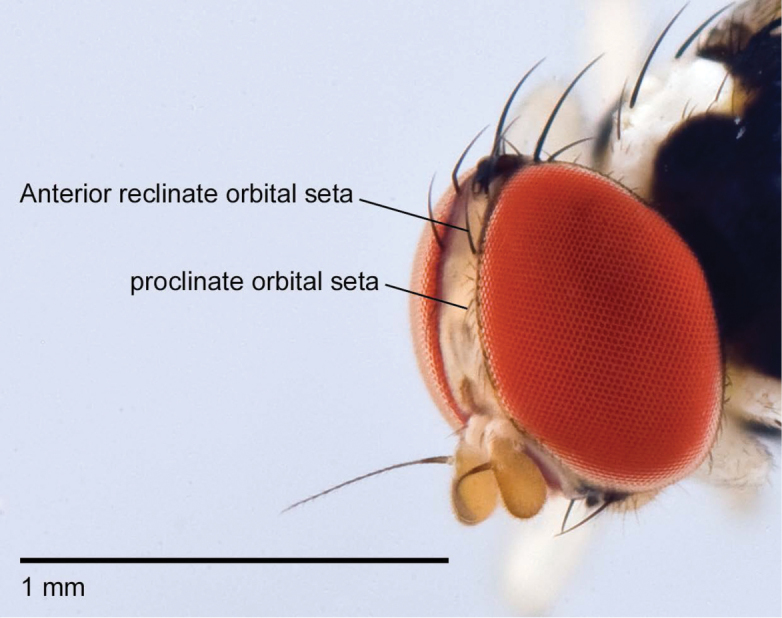
*Acletoxenus* sp. proclinate orbital setae noticeably shorter than the anterior reclinate setae.

The flies were confirmed to belong to *Acletoxenus* by S McEvey (pers. comm.) and G. Bächli (pers. comm.). Specimens representing the Singapore *Acletoxenus* population have proclinate orbital setae that are noticeably shorter than the anterior reclinate setae (Fig. 3). According to the identification key in Malloch (1929; see couplet 1), only two of the four described species of *Acletoxenus* have this trait (*A. indicus*; *A. quadristriatus*), but note that Bächli et al.’s (2004) redescription of *A. formosus* mentions that this species also has noticeably shorter anterior reclinate setae. This means that the bristle character observed in the Singapore specimens only excludes *A. meijerei*. It was hoped that species identification would be possible based on the mesonotum coloration patterns that feature prominently in the taxonomic literature on *Acletoxenus*. However, the Singapore population includes specimens that match the patterns of three of the four described species of *Acletoxenus* (Fig. 2): the *A. quadristriatus* morphotype is only present in males while the other two morphotypes are found in both sexes (Fig. 2). Gender and morphotypes were significantly co-dependant (Fisher’s exact probability test, p-value < 0.01) with the *A. formosus* morphotype being more common in females. An additional character system that is discussed in the literature is the coloration patterns of the abdomen. However, the dorsocentral black mark on the fourth tergite and a much smaller mark of similar shape on the fifth tergite are found in all morphotypes (Fig. 2). The coloration patterns on the remaining tergites are also variable in the Singapore population and range from broadly blackened tergites (Fig. 2A) to reduced spots (Fig. 2B, C). Note that such intraspecific variability has previously been noted for *A. formosus* (Collin 1902, Malloch 1929, Bock 1982) but it is here confirmed for yet another *Acletoxenus* species.

For two reasons, we are confident that this morphological variability in the Singapore population was indeed intraspecific. Firstly, it appears unlikely that more than one species was found on the same hallway of a building on NUS campus. Secondly, COI barcodes were sequenced for two individuals of each sex and morphotype. When these sequences were aligned and compared, the average pairwise distance between the 12 individuals from Singapore was 0.06% which is compatible with intraspecific variability and rarely observed between species of Diptera (Meier 2008, Meier et al. 2008). When the sequences were compared with those in Genbank, the best uncorrected pairwise match was 1.69% (Srivathsan and Meier 2012) and the matching sequence belonged to a specimen from China that was identified as *Acletoxenus indicus* (Accession number: HQ701131.1). The match to a sequence for *A. formosus* (Accession number EF576933.1) was much poorer (11.14%) and is consistent with being interspecific (Meier et al. 2008). No sequences are known for *Acletoxenus quadristriatus* which is only known from Thursday island and was described after *A. indicus*. Overall, there is no described feature that distinguishes the Singapore population from *A. quadristriatus* or *A. indicus* but the latter is hypothesized to have a wide distribution that is compatible with the occurrence of the species in Singapore; i.e., based on overall evidence, we believe that the Singapore population either belongs to *A. indicus* or represents a closely related species because a barcode distance of 1.69% is reasonably common within but also between species given that COI is not directly involved in speciation and only measures time of divergence (Kwong et al. 2012). If *A. indicus* is indeed polymorphic and widespread, it raises the possibility that *A. quadristriatus* could be a junior synonym of *A. indicus*. However, this issue can only be addressed by detailed study of all types. A stumbling block will be the fact that *A. indicus* was described based on a female; i.e., one would have to find a species-specific character in a female that can distinguish this species from all others.

Overall, it is frustrating that despite having obtained considerable amounts of morphological and molecular data, the specimens could not be identified confidently to species. In the case of *Acletoxenus*, it was the widespread use of color pattern characters and a species description based on a female that caused this problem. But identification problems are so common that they play a major role in the decline of natural history research (Tewksbury et al. 2014). Many observations on insects and other animals are made but they are difficult to communicate because the species involved cannot be identified even if a voucher is collected. This problem is particularly severe in the tropics where the species diversity is high (e.g., Basset et al. 2012), most species are undescribed (e.g., Riedel et al. 2010), and many old descriptions are so superficial that they cannot be used for species identification (Meier 2017). Arguably, the best way forward will be higher quality (re)descriptions (Tan et al. 2010, Ang et al. 2013b, Rohner et al. 2014), digital reference collections including types and specimens identified by taxonomic experts (Ang et al. 2013a), and DNA barcodes (Hebert et al. 2003). The latter are becoming sufficiently cost-effective (Wong et al. 2014, Meier et al 2016) that they can become widely available. They can be used to obtain approximate species identifications once more of the fauna is barcoded (Kwong et al. 2012). This can now happen rapidly through low-cost “NGS barcoding” (Meier et al. 2016). Hopefully biologists will start collecting vouchers associated with interesting natural history observations that can be published in journals such as the Biodiversity Data Journal (Smith et al. 2013). The natural history observations can be included in such publications where the video evidence can be embedded in the publication (e.g., Ang et al. 2013b).

### Are *Acletoxenus* Predators?

**Figure 4.**
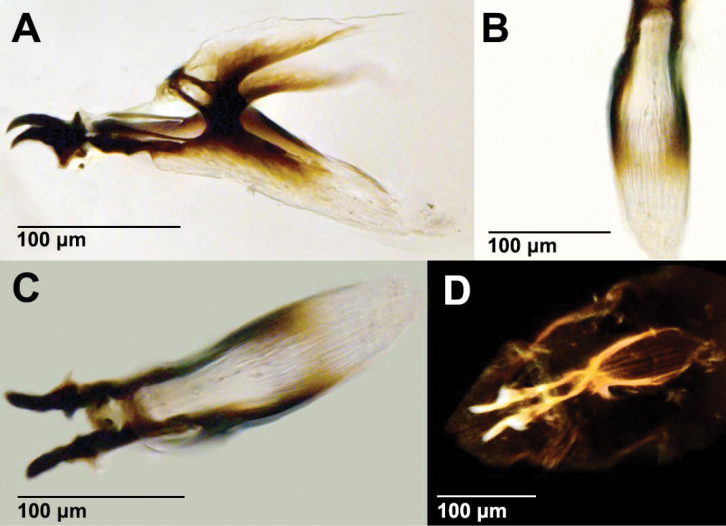
Acletoxenus cf. indicus larvae **A** feeding on whitefly **B** have a green colored body, and **C** are usually covered in whitefly wax and instars **D** SEM Lateral view, and **E** SEM of pseudocephalon with strongly reduced facial mask.

The first video evidence that the larvae are indeed predators of whiteflies is presented here (Movie 1). The larvae move on infected leaves by raising and swinging their anterior end (“pseudocephalons”) from side to side (Movie 2). If no prey is touched, the mouth hooks are used to anchor the anterior end of the larva. After anchoring, the abdominal segments move forward via contraction (Movie 3). However, if prey is touched, the larva uses its mouth hooks to stab a whitefly puparium whose content is then imbibed (Fig. 4A, Movie 2). When a whitefly puparium is empty and gets dislodged from the leaf, it is often glued to the body of the *Acletoxenus* larva using a mucus secreted by the larva (Clausen and Berry 1932, Ashburner 1981). Similarly, whitefly eggs and wax are often found glued to the larva. Overall, the larvae move little and slowly (see Movie 3) and Clausen and Berry (1932) even stated that *Acletoxenus indicus* larvae are largely inactive and never leave the leaf upon which they were born. However, this is not the case for the *Acletoxenus* population in Singapore. Larvae did move to other leaves in order to locate prey, albeit at a very slow speed. All movements (forward or backward) were via peristaltic contractions of the abdominal segments (Movie 3).

**Movie 1.** Acletoxenus cf. indicus: larval predation behavior.

**Movie 2.** Acletoxenus cf. indicus: larval feeding behavior and camouflage.

**Movie 3.** Acletoxenus cf. indicus: larval movements.

**Figure 5.**
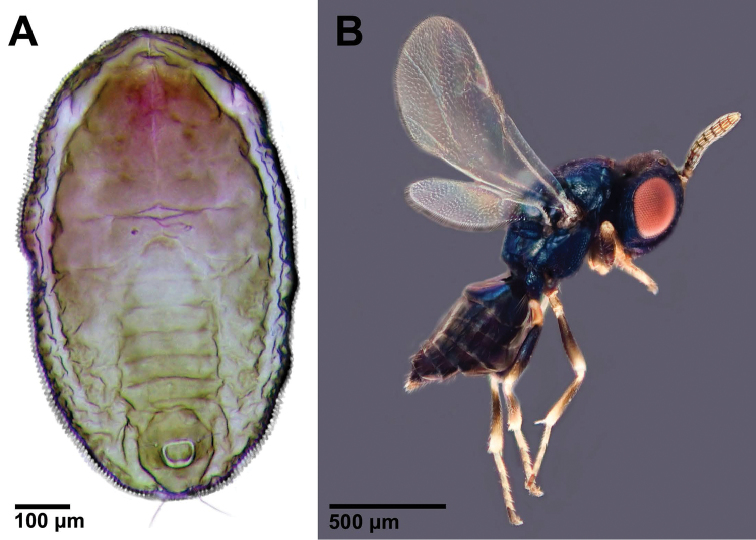
Acletoxenus cf. indicus larval cephaloskeleton **A** lateral view with light microscope **B** ventral view close-up with light microscope **C** ventral view with light microscope, and **D** ventral view with confocal microscope, showing a lack of pharyngeal filter.

**Figure 6.**
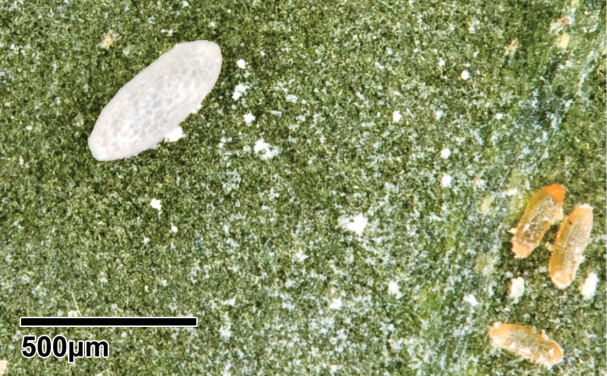
*Drosophila melanogaster* larval cephaloskeleton **A** lateral view with light microscope **B** ventral view close-up with light microscope **C** ventral view with light microscope, and **D** ventral view with confocal microscope, showing a pharyngeal filter.

As discussed in Courtney et al. (2000), most predatory cyclorrhaphan larvae have strongly reduced facial masks that often lack the cirri and oral ridges that are present in saprophagous Cyclorrhapha larvae for rasping and directing bacteria into the mouth opening (Dowding 1967, Roberts 1971). This is also the case for saprophagous ephydroid larvae (Ferrar 1987, Kirk-Spriggs et al. 2002, Wipfler et al. 2013). The pseudocephalon of *Acletoxenus* fits the pattern of a predatory cyclorrhaphan larva. The preoral cavity on the ventral side of the pseudocephalon has few oral ridges flanking the mouth and lacks a well-developed facial mask (absence of cirri; Fig. 4E). The cephaloskeleton of *Acletoxenus* is furthermore semi-translucent and less sclerotized than that of *Drosophila melanogaster* and lacks a pharyngeal filter (Fig. 5) while it was clearly visible for *D. melanogaster* larvae (Fig. 6). Additional adaptations for being a diurnal predator are found on the remaining larval body segments. The larvae are so weakly sclerotized that the internal fat body is visible. It turns from cream-colored in early instars to greenish in third instars (3–4 mm long, 1mm wide; Fig. 4) and thus provides camouflage on leaves (Movie 1 and 2). Camouflage is also the most likely explanation for why the larva collects and glues whitefly wax, egg and puparium onto its body (Fig. 3C; Clausen and Berry 1932, Ashburner 1981). Because pupation of schizophoran flies takes place within the last larval skin, this camouflage extends to the pupal stage of *Acletoxenus* (Fig. 10; ca. 3.3 mm long, 1.3 mm wide) (Fig. 10); the pupal integument remains translucent and reveals the green color of the fat body and later the red eyes of the developing adult (Fig. 10C). The puparia are glued via a flattened ventral surface to leaf surfaces (Clausen and Berry 1932) and the ability to adhere to surfaces is retained even when the puparia are dislodged and placed on moist tissue. The adults emerge by breaking open the distinct lid at the anterior end and leave behind a translucent empty puparium (Clausen and Berry 1932).

**Figure 7.**
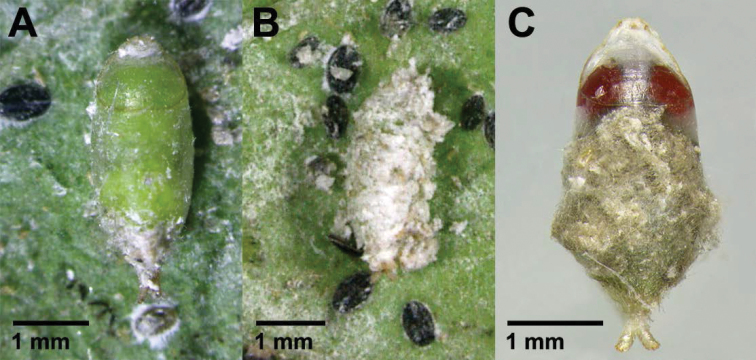
Acletoxenus cf. indicus adult **A** lateral view **B** SEM with proboscis folded in, and **C** SEM showing a typical extended schizophoran proboscis.

In contrast to the larvae that have obvious adaptations for predation, the adults are apparently not predatory. This conclusion is mostly based on observations, but the adults also lack obvious morphological adaptations for predation. For example, the adults have a typical schizophoran proboscis (Colless and McAlpine 1991) with two sponge-like labellar lobes (Fig. 7). Each labellar lobe has six pseudotrachea with likely capillary function (Fig. 7) (Elzinga and Broce 1986).

**Figure 8.**
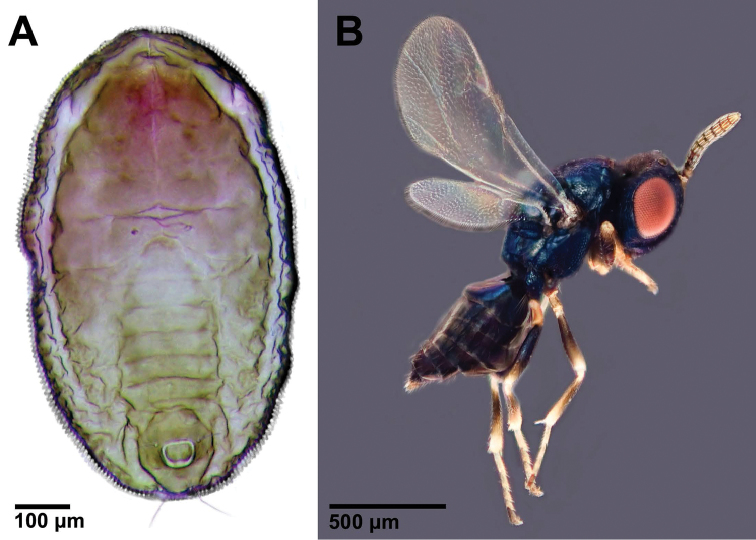
**A** Fourth instar of *Aleurotrachelus trachoides*, the prey of Acletoxenus cf. indicus and **B** adult *Pachyneuron leucopiscida*, the parasite of Acletoxenus cf. indicus.

Prey: *Acletoxenus* larvae belonging to the Singapore population preyed on *Aleurotrachelus trachoides* (Back, 1912) (Fig. 8A) which has fourth instars with dentate margin and a large, setose lingual that expands apically and protrudes beyond the vasiform orifice. These features were used for a preliminary identification but the identity of the prey was also confirmed by D Barro (pers. comm.) and DNA barcodes (99% match to sequence for *Aleurotrachelus trachoides*; accession number KF059957) (Hodges and Evans 2005, Walker 2008). Note that *Aleurotrachelus trachoides*, is a major cosmopolitan pest of commercial plants (Hodges and Evans 2005, Malumphy 2005, Martin 2005, Forest Health 2013 highlights 2014). Thus, Acletoxenus cf. indicus could be considered a potential biological control agent for white flies given that the larvae consume 30 to 40 whitefly puparia during development (Pelov and Trenchev 1973). However, past attempts at using *Acletoxenus* for this purpose have failed (Clausen and Berry 1932, Vayssière 1953) and although the reasons were never fully resolved, it has been suggested that extensive parasitism by Hymenoptera could be a contributing factor (Clausen and Berry 1932, Mentzelos 1967, Pelov and Trenchev 1973). This explanation is partially supported by our data. A high parasitization rate was observed (mean = 43.3%; Table 3) that was caused by a pteromalid wasp (Fig. 8B; Movie 4). This wasp was identified as *Pachyneuron leucopiscida* Mani, 1939. The same species had previously been recorded as a parasitoid of *Acletoxenus indicus* (Gupta & Poorani, 2009). The highest rate of parasitism in the Singapore population was in June while July saw a decrease in both the number of *Acletoxenus* that successfully emerged and the rate of parasitism. As the parasitism rates increased, the population of Acletoxenus cf. indicus declined and it crashed by August.

**Table 3.** Monthly parasitism rates.

Month	Number of parasitized puparium found	Number of non-parasitized puparium found	Percentage of Parasitized Puparium
May 2014	18	21	46.2%
June 2014	16	13	55.2%
July 2014	4	10	28.6%

**Movie 4.** *Pachyneuron leucopiscida* emerging from parasitized Acletoxenus cf. indicus pupa.

### Life cycle of Acletoxenus cf. indicus

**Figure 9.**
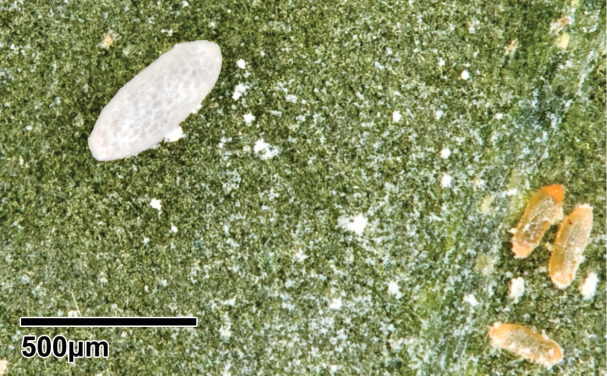
*Acletoxenus* sp. egg (left) found next to whitefly first instars (right).

Acletoxenus cf. indicus’ mean development time in Singapore was 24 days (Table 4). This is similar to the life cycle duration of European *Acletoxenus formosus* whose development time varies from 12 (Frauenfeld 1868) to 27 days (Pelov and Trenchev 1973). The mean lifespan of the adult flies was 12 days (Table 4). The Singapore population of Acletoxenus cf. indicus oviposits in the morning and afternoon and early instars of whiteflies are the initial prey while Clausen and Berry’s (1932) described oviposition by *Acletoxenus indicus* during midday. In Singapore, the eggs were laid singly and the number of eggs oviposited on one leaf varied from one to four. All eggs were white and firmly attached to the abaxial surface of the leaves (Clausen and Berry 1932; Fig. 9). The eggs are approximately 0.45 mm in length and 0.2 mm in width with somewhat indistinct reticulate markings; The eggs of the Singapore population are thus slightly bigger compared to the eggs of *Acletoxenus indicus* in Clausen and Berry (1932; 0.4 mm length).

**Table 4.** Time spent in each life cycle stage of Acletoxenus cf. indicus.

Stage	Mean number of days	Standard deviation
Egg	3.5	1.1
Larva	12.4	2.8
Puparium	8.6	2.4
Adult	12.0	4.8

**Figure 10.**
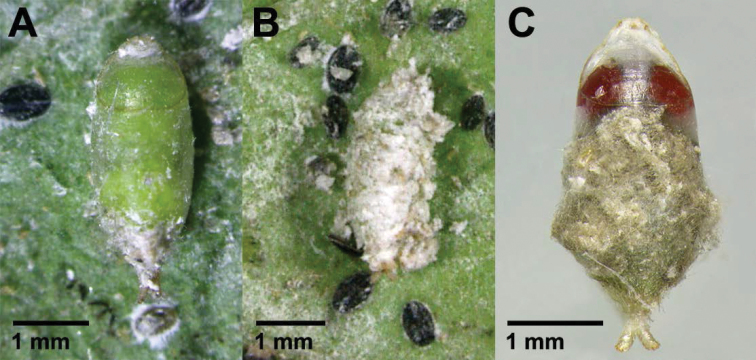
Acletoxenus cf. indicus puparium **A** with green body, is usually **B** covered in whitefly wax and instars, and **C** translucent integument revealing red eyes of the developing adult at later stages.
